# Glucocorticoids and Catecholamines Affect in Vitro Functionality of Porcine Blood Immune Cells

**DOI:** 10.3390/ani9080545

**Published:** 2019-08-12

**Authors:** Lena Reiske, Sonja Schmucker, Julia Steuber, Volker Stefanski

**Affiliations:** 1 Department of Behavioral Physiology of Livestock, Institute of Animal Science, University of Hohenheim, Garbenstr. 17, 70599 Stuttgart, Germany; 2 Department of Cellular Microbiology, Institute of Microbiology, University of Hohenheim, Garbenstr. 30, 70599 Stuttgart, Germany

**Keywords:** pig, stress, immune system, cortisol, adrenaline, noradrenaline, catecholamines, lymphocytes, cytokines

## Abstract

**Simple Summary:**

In modern livestock husbandry, animals may face stressful events like weaning, regrouping, or transportation, all of which can impair animal welfare and health. Research in model organisms has revealed that stress hormones, such as glucocorticoids and catecholamines, strongly modulate the immune system and thus the animals’ ability to fight infections. In the pig, knowledge about this relationship is rare, and results from rodents cannot readily be transferred due to some physiological differences. Therefore, the effects of glucocorticoids and catecholamines on porcine immune cell proliferation and the production of the pro-inflammatory cytokine TNFα were investigated in an in vitro study. Blood was obtained from catheterized pigs to exclude pre-exposure to stress hormones. Glucocorticoids exerted inhibitory effects on both investigated immune functions. Catecholamines, on the other hand, showed diverse effects on lymphocyte proliferation and TNFα production of particular immune cell types. This suggests that studies from model species are not entirely transferrable to pigs. Future research should extend the preliminary findings on cytokine production and focus on the molecular mechanisms and health impacts of stress hormones in pigs.

**Abstract:**

Stress hormones exert important modulating influences on the functionality of immune cells. Despite its major role as a livestock animal and its increasing use as an animal model, knowledge about this relationship in the domestic pig is rare. This study therefore aimed to characterize the effect of glucocorticoids and catecholamines on the proliferation and cytokine production of porcine peripheral blood mononuclear cells (PBMC). Blood was obtained from donor pigs equipped with indwelling catheters to exclude stress hormone exposition before in vitro testing. PBMC were stimulated in the presence of cortisol, adrenaline or noradrenaline at concentrations resembling low to high stress conditions. Proliferation was determined via ^3^H-thymidine incorporation, and TNFα producers were quantified by intracellular cytokine staining. Cortisol led to a decrease in mitogen-induced lymphocyte proliferation and the number of TNFα producing cells. In contrast, catecholamines increased proliferation while exerting repressive or no effects on the number of cytokine producers. Remarkably, in concentrations presumably found in lymphatic tissue in stress situations, noradrenaline suppressed lymphocyte proliferation completely. The shown repressive effects might especially have implications on health and welfare in pigs. The obtained results provide a preliminary database for extended studies on the molecular mechanisms of glucocorticoid and catecholamine actions on porcine immune cells.

## 1. Introduction

The physiological stress response enables the body to cope with threats via predominantly adaptive alterations in cardiac function, energy metabolism and the immune system [1,2,3]. However, if stress exposure lasts for a long time, it can negatively affect animal welfare and health. Chronically elevated levels of stress hormones, namely glucocorticoids (GCs) and the catecholamines (CAs) adrenaline (ADR) and noradrenaline (NA), contribute to an impaired immune function leading to increased risk of infection and reduced animal welfare [4,5]. Efforts to reduce the use of antibiotics in animal husbandry also require a well-functioning immune system and the prevention of stress-induced immunosuppression. For these reasons, it is of utmost importance to understand the actions of the particular stress hormones on different immune functions. So far, this topic has mostly been studied in humans and rodents. It was thus shown that GCs can inhibit important immune functions such as lymphocyte proliferation [6,7] and the production of pro-inflammatory cytokines like TNFα and IFNγ [8,9]. ADR and NA can exert effects similar to cortisol with lower proliferation [10] and cytokine production [11,12]. However, they may also lead to immune activation [13,14], depending on experimental conditions, such as dose or the timing of treatment [15]. 

In modern pig husbandry systems, animals face many potential stressors that can cause a release of GCs and CAs [5,16]. Cortisol (C), as the main GC in pigs, can thus be raised from basal levels of 20–30 ng/mL (8.3 × 10^−8^ M) to a plasma concentration of about 350 ng/mL (9.7 × 10^−7^ M) in highly stressful situations [17,18]. Using blood samples from catheterized pigs and thus avoiding a rapid CA release due to stressful sampling techniques, basal plasma ADR concentrations of approximately 180 pg/mL (10^−9^ M) and NA concentrations of around 325 pg/mL (2 × 10^−9^ M) were found [19]. In acute stress situations, plasma ADR concentrations can range between 700 pg/mL (1.5 × 10^−9^ M) and 100 ng/mL (5.5 × 10^−7^ M), while NA may reach levels between 1700 pg/mL (10^−8^ M) and 300 ng/mL (1.8 × 10^−6^ M) [20,21].

Even though the increase of GCs and CAs upon stressor exposure is well documented in pigs, only a few experiments have studied the functionality of immune cells under the influence of stress hormones in this important livestock species so far. It was shown, for example, that social isolation, weaning, restraint or regrouping led to an increase in endogenic cortisol production, thus resulting in the suppression of lymphocyte proliferation [16,17,22,23,24] and a reduced expression of pro-inflammatory cytokines [21,25,26]. However, it is likely that these immune-modulating effects cannot solely be attributed to cortisol, as a concurrent activation of the sympathetic nervous system (SNS) which leads to the secretion of ADR and NA is probable. Studies that separately examine the effect of stress hormones in pigs are rare, and there are no studies on the specific effects of CAs on the functionality of porcine immune cells. It cannot readily be assumed that the effects of stress hormones observed in rodent studies are the same in pigs, as there are some important anatomical and physiological species differences. For example, the circadian rhythm of the plasma GC concentrations and blood immune cell numbers of rodents are opposite to that of pigs with regard to light and darkness [27,28,29]. Moreover, it is assumed that the porcine hypothalamus–pituitary–adrenal (HPA) axis is less sensitive than its rodent counterpart [30,31] while having ontogenetic similarities to humans [32]. Therefore, it would be premature to assume that findings from rodent studies are fully transferable to pigs. To get a better understanding of stress-induced immunomodulation in pigs, more studies are needed. A useful first approach is to examine the actions of the different stress hormones separately in a controlled in vitro environment, where conditions can be standardized and disruptive factors can be minimized compared to in vivo models.

The aim of the present study was thus to investigate the impact of different infra-to-supraphysiological concentrations of cortisol, adrenaline and noradrenaline on porcine lymphocyte proliferation in vitro. In addition, we also examined the effect of the three stress hormones on the number of TNFα producing immune cells among different leukocyte subsets.

## 2. Materials and Methods 

### 2.1. Animals and Sampling

All procedures were conducted according to the ethical and animal care guidelines and approved by the local authority for animal care and use (Regional Council Stuttgart, Germany; ethical approval code: V324/15TH). In total, 32 castrated male pigs (German Landrace x Pietrain, 7–10 months old, body weight range 90–120 kg), divided into three consecutive experimental trials with 10–12 animals each, were available as blood donors for this study. Blood from each individual donor pig was used only once for each tested immunological parameter. The barrows were housed individually in pens (7 m²) with sight and tactile contact through the bars. Concentrate (1.3–1.5 kg/meal, ME 12 MJ/kg) was fed twice daily (0730 and 1500), and pigs had ad libitum access to water and hay. Pens were cleaned daily after feeding in the morning and littered with dust-free wood shavings. Light was turned on from 0630 until 2030. Since blood sampling methods including fixation by nose snare or obtaining blood at slaughter already resemble stressful conditions and thus compromise a controlled investigation of defined hormone concentrations, pigs were equipped with indwelling vein catheters via Vena cephalica cannulation. Surgery was performed as published by Kraetzl and Weiler [33] with modifications described in Engert et al. [29] at least 14 d before sampling. All animals were thoroughly habituated to human handling to ensure stress free blood sampling via the vein catheters. Blood (10 mL per animal) was collected into lithium heparin tubes (Sarstedt, Nümbrecht, Germany) at 0830. 

### 2.2. Isolation of Peripheral Blood Mononuclear Cells (PBMC)

Porcine peripheral blood mononuclear cells (PBMC) were separated using Leucosep^TM^ centrifuge tubes (Greiner Bio-One, Frickenhausen, Germany) and Biocoll (density: 1.077 g/mL, Biochrom, Berlin, Germany) according to the manufacturer’s protocol with the following modifications: After separation, cells were washed in PBS (Biochrom) supplemented by 2 mM EDTA (Sigma-Aldrich, Taufkirchen, Germany) and subsequently in RPMI 1640 supplemented by 5% inactivated fetal calf serum (FCS) and 50 µg/mL of gentamycin (all Biochrom). PBMC were then suspended in RPMI 1640 supplemented with 10% FCS and 50 µg/mL gentamycin, and cell concentration was measured using a Z2 Coulter Counter (Beckman Coulter, Krefeld, Germany). 

### 2.3. Lymphocyte Proliferation Assay

Using the PBMC of 20 donor pigs from Trials 1 and 2, a mitogen-induced lymphocyte proliferation assay was performed as previously described [34], including a dilution series of each investigated hormone. In brief, 1.5 × 10^5^ of PBMC were seeded per well and stimulated with 5 µg/mL concanavalin A (ConA) or 5 µg/mL pokeweed mitogen (PWM, both Sigma-Aldrich) of left without stimulation. Stimulated samples were left without hormones or additionally supplemented with either C, NA or ADR in final concentrations of 10^−10^, 10^−9^, 10^−8^, 10^−7^, 10^−6^, or 10^−5^ M, covering miscellaneous possible plasma concentrations from calmness to high stress. All treatments were done in triplicates. A second experiment with the PBMC of 12 barrows from Trial 3 was conducted including only NA in concentrations of 10^−6^, 10^−5^, and 10^−4^ M, resembling the presumed milieu around noradrenergic nerve endings in lymphatic tissues [35,36]. Cells were incubated at 39 °C and 5% CO_2_ for 48 h, after which 0.25 µCi ^3^H-thymidine were added for a further 24 h. Cells were harvested on glass fiber filters (Sigma-Aldrich), and the incorporated amount of radioactivity was measured in counts per minute (cpm) by a liquid scintillation analyzer (PerkinElmer, Rodgau, Germany). For statistical analysis, the cpm of the unstimulated triplicates were subtracted from the stimulated ones to obtain the ∆cpm. In the NA high-dose experiment, cpm were used for data analysis, as the highest NA dose led to negative ∆cpm values.

### 2.4. Intracellular Cytokine Staining

For the investigation of the effects of stress hormones on the number of immune cells producing pro-inflammatory cytokines, an intracellular staining technique was conducted with the blood of 23 pigs from Trials 2 and 3. After separation, 10^6^ of PBMC were transferred into sterile polystyrene tubes and, after the addition of either stress hormone in high (10^−6^ M) or moderate (10^−8^ M) concentrations or no hormone at all, cells were either left unstimulated or stimulated with 5 µg/mL PWM, which was found best suitable to elicit TNFα production without overstimulation, ensuring a sufficient sensitivity to hormone effects in own preceding experiments. To inhibit the secretion of cytokines, 1 µg/mL of brefeldin A was added. Cells were incubated for 4 h (39 °C, 5% CO_2_) and subsequently fixated with a formaldehyde buffer (PBS, 2mM EDTA, 0.5% FCS, 0.5% Roth-Histofix formaldehyde, Karl Roth GmbH, Karlsruhe, Germany) for 20 min at room temperature. Then, cells were permeabilized using a saponin buffer (PBS, 2mM EDTA, 0.5% FCS, 0.05% saponin) and stained (15 min, 6 °C) with the following antibodies: CD3ε-biotin (clone PPT3, Acris Antibodies, Herford, Germany) and streptavidin-V500, CD4-PerCP-Cy5.5 (clone 74-12-4), CD8α-AlexaFluor 647 (clone 76-2-11), IFNγ-PE (clone P2G10, all BD Biosciences, NJ, USA) and TNFα-PacificBlue (clone Mab11, Biolegend, San Diego, CA, USA). Afterwards, cells were washed in saponin buffer and resuspended in PBS + 1 % FCS. Analysis was performed using a FACSCanto II^TM^ flow cytometer (BD Biosciences) with the software BD FACSDiva^TM^ by evaluating the percentage of cytokine-producing cells per population (10^5^ events/sample). Populations were differentiated based on surface marker expression into: Cytotoxic T cells (CTL; CD3^+^CD4^−^CD8α^high^, ~10^4^ events), γδ T cells (CD3^+^CD4^−^CD8α^−/low^, ~2 × 10^4^ events), naive T helper (T_H_) cells (CD3^+^CD4^+^CD8α^-^, ~10^4^ events), antigen-experienced (Ag-exp.) T_H_ cells (CD3^+^CD4^+^CD8α^+^, ~10^4^ events) and natural killer (NK) cells (CD3^−^CD4^−^CD8α^+^, ~10^4^ events). Due to a high background of IFNγ in the unstimulated samples, only the number of total TNFα producers were investigated and used for statistical analysis. For technical reasons, the intracellular staining of monocytes was conducted with deep-frozen PBMC. Therefore, the PBMC of 6 animals of Trial 3 stored at −80 °C in DMSO (Sigma-Aldrich) were thawed in RPMI-10 at 37 °C and washed twice in RPMI-5 before determination of cell concentration. Stimulation was conducted analogous to the first trial but with 1 µg/mL lipopolysaccharide (LPS; Sigma-Aldrich) used as stimulant. Cells were then stained with the antibodies CD172a-PE (clone 74-22-15A, BD Biosciences) and TNFα-PacificBlue (clone Mab11, Biolegend). 5 × 10^4^ events per sample were recorded, and monocytes were defined as CD172a^+^ cells (~2 × 10^3^ events).

### 2.5. Statistical Analysis

Data were analyzed using SAS Version 9.4 (SAS Institute Inc., Cary, NC, USA). We used the MIXED procedure of SAS with degrees of freedom determined by the Kenward–Roger method [37]. Linear mixed-effect models included the factor treatment (addition of no hormone or different concentrations of C, NA, or ADR) as a fixed effect and individual (1–20, 1–12, 1–23), sampling date, and trial (1–3), as well as their interactions, as random effects. Normality and variance homogeneity were confirmed by visually checking normal probability plots and plots of fitted values versus residuals [38]. If necessary, square root or logarithmic transformation was performed. For all comparisons, *p* < 0.05 was considered significant. All results are presented as LS-means + standard error of the mean (SEM).

## 3. Results

### 3.1. Lymphocyte Proliferation

**Figure 1:**
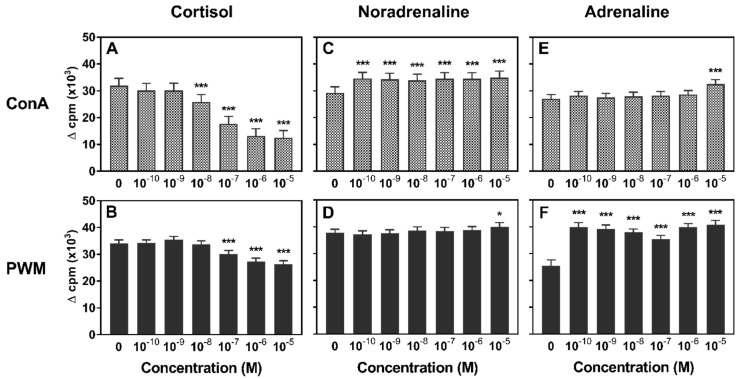
Lymphocyte proliferation after incubation with cortisol (**A**,**B**), noradrenaline (**C**,**D**) or adrenaline (**E**,**F**) (10^−10^–10^−5^ M) and one of the mitogens concanavalin A (ConA) (**A**,**C**,**E**) or pokeweed mitogen (PWM) (**B**,**D**,**F**) in vitro (*n* = 20). Data are presented as lsmeans + standard error of the mean (SEM) of Δcpm (counts per minute) of the untransformed data. Asterisks indicate significant differences between treatment and hormone-free control (0): * *p* ≤ 0.05; *** *p* < 0.001.

To investigate stress hormone effects on lymphocyte proliferation, we tested a wide range of concentrations in a mitogen-induced proliferation assay. Compared to the hormone-free control, cortisol caused a significant reduction of lymphocyte proliferation in a dose-dependent manner. When PBMC were stimulated with ConA, this inhibitory effect occurred at a concentration of 10^−8^ M and higher, whereas the proliferation of PWM-stimulated PBMC was first inhibited upon addition of 10^−7^ M cortisol (Figure 1a,b). In contrast, catecholamines generally had an enhancing impact on lymphocyte proliferation, but the magnitude of the effect of adrenaline or noradrenaline action was dependent on CA dose and mitogen (Figure 1c–f). Noradrenaline increased ConA-induced proliferation in all tested concentrations (Figure 1c). An enhancing effect could also be observed on PWM-stimulated PBMC proliferation but at a lower magnitude and only for the highest tested concentration of 10^−5^ M. Similarly, adrenaline led to a higher proliferation of mitogen-stimulated PBMC, but, here, the effect was much more pronounced for PWM than for ConA. If stimulated with PWM, all investigated concentrations enhanced lymphocyte proliferation significantly (Figure 1f), while ConA-stimulated proliferation was enhanced only for 10^−5^ M ADR (Figure 1e). 

**Figure 2:**
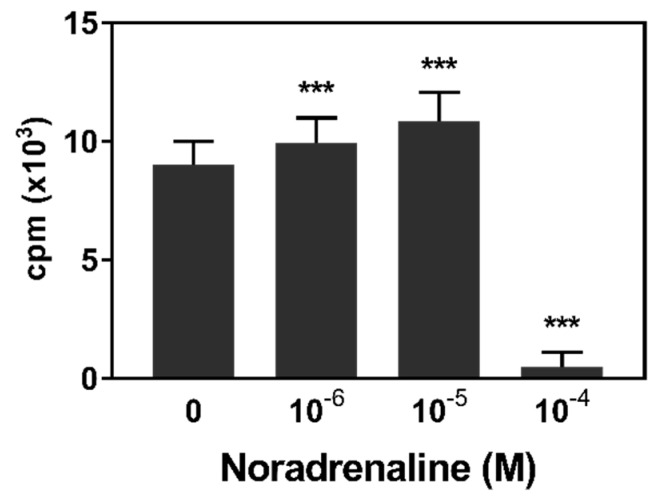
Lymphocyte proliferation after incubation with noradrenaline (10^−6^–10^−4^ M) and the mitogen pokeweed mitogen in vitro (*n* = 12). Data are presented as lsmeans + SEM of cpm (counts per minute) of the untransformed data. Asterisks indicate significant differences between treatment and hormone-free control (0): *** *p* < 0.001**.**

Beside production by the adrenal medulla and release into the blood stream, noradrenaline is also widely used as a neurotransmitter in the SNS. It can thus reach high local concentrations at sympathetic nerve endings, which are present in abundance in lymphoid tissues [28,29]. Therefore, a further experiment was conducted using higher NA concentrations (Figure 2). Again, NA at concentrations of 10^−6^ and 10^−5^ M caused an increase of PWM-induced proliferation. A higher NA concentration of 10^−4^ M, however, led to a drastic reduction of cpm.

### 3.2. Intracellular Cytokine Staining

**Figure 3:**
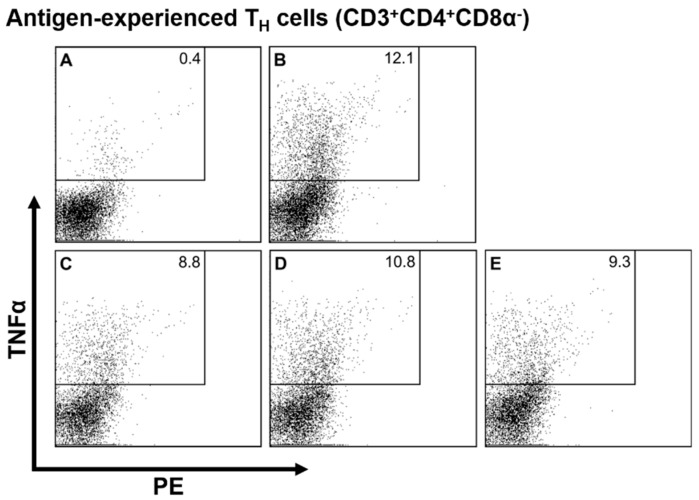
Representative plots of TNFα producers among antigen-experienced T helper (T_H_) cells. Porcine peripheral blood mononuclear cells (PBMC) were stimulated with pokeweed mitogen and antigen-experienced T_H_ cells were discriminated based on surface marker expression. TNFα is plotted on the y axis against the PE channel on the x axis. TNFα-positive cells are shown in the rectangular gates, numbers in the corner indicate the percentage of TNFα producers among antigen-experienced T_H_ cells. Letters in the upper left corner indicate the treatment of the sample: A = No stimulation; B = Stimulated hormone-free control; C = Cortisol (10^−6^ M); D = Noradrenaline (10^−6^ M); E = Adrenaline (10^−6^ M).

To get a more differentiated picture of the impact of stress hormones on immune cell activation, we used an intracellular staining technique which allowed us to quantify TNFα producers separately for different immune cell types. The results of linear mixed model analysis are shown in Table 1, and representative dot plots (i.e., antigen-experienced T_H_ cells) are shown in Figure 3. In all investigated leukocyte subsets except NK cells, cortisol at a concentration of 10^−6^ M decremented the number of TNFα producers (Table 1, Figure 3C), while lower cortisol concentrations of 10^−8^ M had no effect. For noradrenaline, on the other hand, neither of the tested concentrations had a significant impact on TNFα producing cells in any of the investigated cell types. Similar to cortisol, adrenaline reduced the number of cytokine-producing cells in some leukocyte populations. TNFα producers were reduced among γδ T cells and monocytes if ADR was added at a concentration of 10^−6^ M. The addition of ADR at the low concentration of 10^−8^ M had no significant effect on any of the investigated subsets.

## 4. Discussion

In this study, we found inhibitory as well as stimulatory effects of stress hormones on the proliferative capacity of porcine lymphocytes, depending on the hormone and concentration applied. This study also provides preliminary data on the effects of stress hormones on cytokine producing cells. Cortisol caused a significant reduction of lymphocyte proliferation in a dose-dependent manner, which is in accordance with results from social stress experiments. Deguchi and Akuzawa [17], for example, reported that, after regrouping, piglets showed elevated blood cortisol concentrations of 2 × 10^−7^ M accompanied by a reduced lymphocyte proliferation. In the present study, this immunosuppressive effect could be confirmed by in vitro cultivation with a similar amount of cortisol, proving the suitability of the chosen model. If stimulated with PWM in the presence of 10^-8^ M cortisol, proliferation was still on the same level as the hormone-free control. In mouse experiments, this concentration sufficed to inhibit lymphocyte functionality [39], which may be another hint that the porcine HPA axis is less GC-sensitive than their murine counterpart. 

In contrast to cortisol, which takes a few minutes to rise and is responsible for the detrimental immune outcome in chronic stress situations, catecholamines are released into the blood circulation within seconds after a stressor [3,40]. As reviewed by Elenkov et al. [15], CAs can have inhibitory or stimulatory effects on immune cell functionality, depending on immune cell type, adrenoceptor (AR) type and abundance on these cells, as well as the localization and timing of the CA release. The immunomodulatory properties of ADR and NA were already investigated by Hadden et al. in the 1970s in an in vitro experiment on the phytohemagglutinin-induced proliferation of human lymphocytes [41]. Similar to the data presented here, NA had a β-AR-mediated inhibitory effect if 10^−4^ M were added, whereas lower concentrations of 10^−7^ M stimulated proliferation via α-ARs. An enhanced proliferation was also found in a study with murine B cells stimulated under the influence of 10^−6^–10^−5^ M NA [13]. For adrenaline, Hadden et al. found no effect on lymphocyte proliferation and concluded that stimulating α- and inhibiting β-adrenergic actions nullified each other. In contrast, adrenaline also had an enhancing effect on proliferation in the present study, particularly distinct if PWM was used for stimulation. This seems to indicate that NA effects on proliferation might be mediated by similar mechanisms in human, murine, and porcine lymphocytes, while ADR seems to work differently in pigs, possibly caused by a shifted AR-ratio. In other species, stimulatory α_2_-ARs on B and T cells are upregulated under certain disease states [42]. Future research into type and quantity of ARs on porcine immune cells could reveal whether they express higher numbers of α_2_-ARs than other species even under healthy conditions.

In order to get a more detailed picture on which cell types become activated or suppressed under the influence of stress hormones, we assessed cytokine production on the cellular level. In pigs, the T_H_1/T_H_2 paradigm is not very well investigated, and recent studies have indicated that some cytokine functions are different in pigs compared to other species. The classical T_H_2 cytokine IL-4 does not fulfill this role in pigs, as it suppresses both T_H_1 and T_H_2 immunity including antibody secretion by B cells [43,44]. There are hints that instead of shifting the immune response from T_H_1 to T_H_2, GCs seem to be generally inhibitory in pigs [45]. Furthermore, IFNγ, which is usually increased in a T_H_1 immune response, can be constantly produced in comparatively high concentrations in pigs [46] and is less sensitive to cortisol-mediated inhibition than other cytokines [47,48]. Because pre-tests did not reveal detectable IL-4 amounts upon mitogenic stimulation and IFNγ production was hardly overcoming background production, the effects of stress hormones on cytokine production of porcine PBMC were solely characterized by analysis of TNFα production in the present study. Though in varying amounts, this cytokine is produced by many porcine immune cell types, i.e., monocytes/macrophages, NK cells, γδ T cells, CTL and T_H_ cells, and is thus a good pan-marker of pro-inflammatory activation [43,49,50,51]. 

We discovered that the cortisol-mediated inhibition of immune cell activity did not only result in a reduced lymphocyte proliferation but also in lower numbers of cells producing TNFα in all investigated subsets except NK cells. This is in accordance with studies in humans and rodents, where GCs generally had a suppressive effect on the production of pro-inflammatory cytokines [8,9,52]. In the present study, cell populations of both innate and adaptive immune response were affected, which may have negative effects on the acute response to pathogens as well as memory formation.

While having dose-dependent inhibitory or stimulatory effects on proliferation, none of the tested concentrations of NA had a significant effect on the number of TNFα producers in any of the investigated subsets. Other studies have reported inconsistent results regarding the impact of NA on TNFα production. Some have found an increased number of TNFα producers in human lymphocytes [14], whereas others have observed a decrease of TNFα production in human whole blood cultures [53,54]. This again emphasises the diversity of possible CA actions, and with the present limited data, it would thus be premature to make conclusions about the underlying molecular mechanisms. However, some substantiated speculations about possible pathways in comparison to literature can be made. Presuming that pigs, similarly to humans, have a low number of ARs on T_H_ cells in comparison to other immune cells [55], the absent responsiveness of the two T_H_ cell subsets toward both NA and ADR could be explained. Considering the high number of ARs on NK cells in other species, it is somewhat surprising that cytokine production of porcine NK cells was influenced by none of the stress hormones tested. In other species, NK cell activity is a very sensitive indicator of catecholamine action via β-adrenergic mechanisms [56] and GC-induced immunosuppression [57]. This discrepancy remains subject to future studies.

Interestingly, although PWM-induced proliferation increased significantly under ADR influence, the number of TNFα producers among γδ T cells and monocytes decreased if cultured with 10^-6^ M ADR, while other populations remained unaffected. These puzzling results might be explained by a possible particular action of ADR on regulatory T cells (Tregs). Using human PBMC from breast cancer patients, Zhou et al. [58] demonstrated that an in vitro culture in the presence of ADR resulted in an increased Treg proliferation. If porcine Tregs show the same effect under ADR treatment, their proliferation might also have been enhanced in the present study. As Tregs have an inhibitory effect, especially on the functionality of antigen presenting cells including monocytes [59], they might have hampered TNFα production in monocytes as well as their ability to induce cytokine production in other populations. To verify if Tregs are a special target of ADR action in the pig, studies investigating lymphocyte proliferation on the single cell level using fluorescent dyes, including markers for Foxp3 expression and the analysis of IL-10 concentration in cell culture supernatants, should be conducted. The inhibition of γδ T cells by ADR deserves special emphasis, as their numbers in porcine blood are higher than in mice and humans [60] and they are of great importance, especially in growing pigs [16]. The downregulation of pro-inflammatory cytokines in γδ T cells might therefore have implications for their own role in the early immune response to infections [60], as well as their regulatory function [61] on other immune cells in acute stress situations. 

## 5. Conclusions

Especially in the light of growing public interest in animal welfare and stress assessment in livestock, this study contributes to a better understanding of stress-induced immunomodulation in pigs. The results provide further indications of the immunosuppressive effects of glucocorticoids on immune cell functionality found in previous studies in pigs and other species. The observed impairment of both innate and adaptive immune cells might have implications on various functions like the elimination of infected cells by CTLs, the induction of B cells by T_H_ cells, or phagocytosis by macrophages. In addition, catecholamine-mediated inhibitory as well as stimulatory immunomodulation was shown for the first time in pigs, thus letting this serve as a preliminary work for the future assessment of molecular mechanisms of stress hormone actions in pigs. Beside further functional parameters, the number and distribution of the distinct glucocorticoid and adrenoceptor types on different immune cell populations or the effect of receptor blockers should be investigated.

## Figures and Tables

**Table 1:** Frequency of TNFα producing cells (%) after stimulation in the presence of cortisol, noradrenaline or adrenaline.

Frequency (%)	Control	Hormone						Pooled SEM	Treatment *p*-Value
		Cortisol		Noradrenaline		Adrenaline			
		10^−8^ M	10^−6^ M	10^−8^ M	10^−6^ M	10^−8^ M	10^−6^ M		
Naive T_H_ cells ^†^	1.37	1.31	1.07 ***	1.37	1.32	1.31	1.31	0.48	<0.001
Ag-exp. T_H_ cells	12.36	12.20	10.09 ***	12.48	11.92	11.89	11.60	1.99	<0.001
Cytotoxic T cells	2.42	2.46	1.93 ***	2.54	2.41	2.39	2.25	0.67	<0.001
γδ T cells ^‡^	1.34	1.31	1.08 ***	1.34	1.27	1.25	1.00 ***	0.08	<0.001
NK cells ^‡^	4.32	4.47	3.82	4.18	3.95	4.46	3.94	0.85	0.307
Monocytes ^†^	26.35		22.15 ***		27.00		23.96 *	2.62	<0.001

Cells were stimulated with pokeweed mitogen (lines 1–5) or lipopolysaccharide (line 6). Data are shown as least-square means with pooled standard error of the mean (SEM). *p-*values indicate a significant effect of the treatment. Data that required ^†^logarithmic or ^‡^ square root transformation are reported on the original scale after back transformation. Asterisks indicate a significant effect of the respective hormone treatment compared to the stimulated hormone-free control: * *p* ≤ 0.05; *** *p* < 0.001.
